# Physiological relation between respiration activity and heterologous expression of selected benzoylformate decarboxylase variants in *Escherichia coli*

**DOI:** 10.1186/1475-2859-9-76

**Published:** 2010-10-19

**Authors:** Thomas G Palmen, Jens Nieveler, Bettina Frölich, Wiltrud Treffenfeldt, Martina Pohl, Jochen Büchs

**Affiliations:** 1 AVT - Aachener Verfahrenstechnik, Biochemical Engineering, RWTH Aachen University, Worringerweg 1, D-52074 Aachen, Germany; 2 Institute of Molecular Enzyme Technology, Heinrich-Heine University Düsseldorf, Research Centre Jülich, D-52426 Jülich, Germany; 3 Bioprocess R&D, Dow AgroSciences LLC, Indianapolis, USA; 4 Current Address: Institute of Biotechnology 2, Forschungszentrum Jülich GmbH, D-52425 Jülich, Germany

## Abstract

**Background:**

The benzoylformate decarboxylase (BFD) from *Pseudomonas putida *is a biotechnologically interesting biocatalyst. It catalyses the formation of chiral 2-hydroxy ketones, which are important building blocks for stereoselective syntheses. To optimise the enzyme function often the amino acid composition is modified to improve the performance of the enzyme. So far it was assumed that a relatively small modification of the amino acid composition of a protein does not significantly influence the level of expression or media requirements. To determine, which effects these modifications might have on cultivation and product formation, six different BFD-variants with one or two altered amino acids and the wild type BFD were expressed in *Escherichia coli *SG13009 pKK233-2. The oxygen transfer rate (OTR) as parameter for growth and metabolic activity of the different *E. coli *clones was monitored on-line in LB, TB and modified PanG mineral medium with the Respiratory Activity MOnitoring System (RAMOS).

**Results:**

Although the *E. coli *clones were genetically nearly identical, the kinetics of their metabolic activity surprisingly differed in the standard media applied. Three different types of OTR curves could be distinguished. Whereas the first type (clones expressing Leu476Pro-Ser181Thr or Leu476Pro) had typical OTR curves, the second type (clones expressing the wild type BFD, Ser181Thr or His281Ala) showed an early drop of OTR in LB and TB medium and a drastically reduced maximum OTR in modified PanG mineral medium. The third type (clone expressing Leu476Gln) behaved variable. Depending on the cultivation conditions, its OTR curve was similar to the first or the second type. It was shown, that the kinetics of the metabolic activity of the first type depended on the concentration of thiamine, which is a cofactor of BFD, in the medium. It was demonstrated that the cofactor binding strength of the different BFD-variants correlated with the differences in metabolic activity of their respective host strain.

**Conclusions:**

The BFD-variants with high cofactor binding affinity (wild type, His281Ala, Ser181Thr) obviously extract thiamine from the medium and bind it tightly to the enzyme. This might explain the hampered growth of these clones. In contrast, growth of clones expressing variants with low cofactor binding affinity (Leu476His, Leu476Pro, Leu476Pro-Ser181Thr) is not impaired. Leu476Gln has an intermediate cofactor binding strength, thus, growth of its host strain depends on the specific cultivation conditions. This paper shows that slight differences of the amino acid composition can affect protein expression and cultivation and might require an adaptation of media components. Effects such as the observed are hardly foreseeable and difficult to detect in conventional screening processes. Via small scale experiments with on-line measurements in shake flasks such effects influencing the cultivation and product formation can be detected and avoided.

## Background

Biocatalysts, i. e. whole cell systems or enzymes have found increased use in industrial biotechnology. To further increase the number of industrial biotechnological processes, new biocatalysts are needed. By utilizing the existing biodiversity [1], enzymes from microorganisms found in nature are screened to find suitable biocatalysts for such processes. Besides these naturally occurring sources of potential enzymes, techniques such as directed evolution [2] are applied. With this technique, mutations in selected genes can be generated. If applied to enzymes, single or few amino acids in these enzymes can be exchanged, resulting in different enzyme variants. In this way, clone libraries of microorganisms harbouring the genes for different variants are created which are screened for desired attributes, e. g. improved enzyme activity, thermostability or stereoselectivity.

One enzyme with an already high stereoselectivity [3] is the benzoylformate decarboxylase from *Pseudomonas putida*. It is named BFDC in recent publications. In this work, we stick to the older abbreviation BFD to be consistent with our previous publications [4-6]. While its main reaction, the non-oxidative decarboxylation of benzoylformate is part of the mandelate biosynthetic pathway [7-9], the physiological function of the biotechnologically interesting side reaction, the carboligation [10] is unknown. In this side reaction, BFD catalyses the formation of chiral 2-hydroxy ketones from benzaldehyde or benzaldehyde derivates as acyl donors and acetaldehyde as the acyl acceptor [11]. 2-Hydroxy ketones are important structural subunits in many biologically active natural products and are also important building blocks for stereoselective syntheses [3], e. g. for the synthesis of ephedrine or bupropion [12]. Depending on the acyl donor, the enantiomeric excess of the product synthesised by BFD catalysis varies as well as the amount of converted substrate in a given time [3,11]. To improve the carboligase activity of BFD, a combination of directed evolution and site-directed mutagenesis has been applied [4]. Upon induction of protein expression, these respective recombinant *E. coli *clones expressed different BFD-variants. Other attempts aimed to alter the substrate specificity of BFD [5,13] and to improve the stereoselectivity of BFD for different substrates [14].

Kensy et al. [15] showed that the induction of cultures can affect the growth of the applied microorganisms. Therefore, expressing different BFD-variants might result in different kinetics of the metabolic activity of the applied strains. Hence, in screening processes, the growth of the cultures has to be monitored. Without appropriate measurement systems, endpoint measurements or costly sampling during cultivation have to be conducted in screening processes. Evaluation of the cultivation performance and product formation on the basis of these few data is problematic. In this study, the RAMOS (Respiratory Activity MOnitoring System) was applied to measure the oxygen transfer rate (OTR) on-line as a parameter for the growth and metabolism of the investigated organisms. In this work, the thiamine auxotroph strain *E. coli *SG13009 pKK233-2 was applied to express different BFD-variants. Thiamine auxotroph strains, such as *E. coli *DH5α*, E. coli *JM109 or *E. coli *M15 are routinely utilized in laboratories all over the world. They are also used to express thiamine dependent enzymes [16-19].

The aim of this study is to show the physiological relation between the respiration activity and the heterologous expression of selected BFD-variants under different culture conditions. This example should increase the awareness for effects that can occur during cultivation and that may influence the expression of the product and the cultivation itself.

## Methods

### Microorganism

Different recombinant clones of the thiamine auxotroph strain *E. coli *SG13009 (Qiagen, Hilden, Germany) were used for the experiments, containing the plasmid pKK233-2 (Pharmacia, Uppsala, Sweden) with the genes encoding different benzoylformate decarboxylase (BFD) variants, including the information for a C-terminal His_6_-tag. The applied expression system has a tightly regulated expression of the BFD variants to prevent unwanted expression without the addition of the inducer IPTG. The wild type-BFD originates from *Pseudomonas putida *[4]. Upon induction with IPTG, the clones produce the wild type BFD or BFD variants that differ in one or two amino acids. Table 1 shows the applied clones and the used abbreviations. The genotype of the applied strain *E. coli *SG13009 is Nal^S^, Str^S^, Rif^S^, Thi^-^, Lac^-^, Ara^+^, Gal^+^, Mtl^-^, F^-^, RecA^+^, Uvr^+^, Lon^+^.

**Table 1:** Applied clones and abbreviations

clone	abbreviation
*Escherichia coli *SG13009:pKK233-2-BFD-wt-His_6_	wt

*Escherichia coli *SG13009:pKK233-2-BFD-His281Ala-His_6_	His281Ala

*Escherichia coli *SG13009:pKK233-2-BFD-Leu476Gln-His_6_	Leu476Gln

*Escherichia coli *SG13009:pKK233-2-BFD-Leu476His-His_6_	Leu476His

*Escherichia coli *SG13009:pKK233-2-BFD-Leu476Pro-His_6_	Leu476Pro

*Escherichia coli *SG13009:pKK233-2-BFD-Leu476Pro-Ser181Thr-His_6_	Leu476Pro-Ser181Thr

*Escherichia coli *SG13009:pKK233-2-BFD-Ser181Thr-His_6_	Ser181Thr

To prepare stock cultures, precultures of *E. coli *SG13009 pKK233-2 clones in LB medium were made. After reaching an optical density (OD) of 3, glycerol solution was added as a cryoprotective, resulting in a final glycerol concentration of 300 g/L. The cultures were stored at -20°C in 1 mL aliquots in cryo-vials.

### Media

*E. coli *SG13009 pKK233-2 was cultivated in buffered LB medium with 10 g/L glycerol. It consists of 5 g/L yeast extract, 10 g/L tryptone, 12.54 g/L K_2_HPO_4 _and 2.31 g/L KH_2_PO_4_. Another complex medium, TB medium, was also applied for the cultivations. It consists of 24 g/L yeast extract, 12 g/L tryptone, 12.54 g/L K_2_HPO_4_, 2.31 g/L KH_2_PO_4 _and 5 g/L glycerol. Furthermore, modified PanG mineral medium [20] was used for the cultivations. It consists of 1.6 g/L NaH_2_PO_4_*H_2_O, 3.2 g/L KH_2_PO_4_, 2.6 g/L K_2_HPO_4_, 0.2 g/L NH_4_Cl, 2.0 g/L (NH_4_)_2_SO_4_, 0.6 g/L MgSO_4_, 0.2 g/L CaCl_2_*H_2_O, 5 g/L glycerol and 1 mL/L trace element solution. The trace element solution consists of 5 mL/L H_2_SO_4 _(conc.), 6 g/L CuSO_4_*5H_2_O, 0.08 g/L KI, 3 g/L MnSO_4_*H_2_O, 0.3 g/L Na_2_MoO_4_, 0.02 g/L H_3_BO_3_, 0.5 g/L CoCl_2_, 20 g/L ZnCl_2 _and 65 g/L FeSO_4_*7H_2_O. The pH of all media was adjusted to 7.0. Furthermore, 0.1 g/L ampicillin was added to each medium. As the applied strain has a neomycin/kanamycin selection marker, additionally 0.05 g/L neomycin was added to each medium. Although *E. coli *SG13009 pKK233-2 is a thiamine auxotroph strain, no additional thiamine was added to the complex LB and TB medium, according to the literature [21-23]. For the cultivations with additional thiamine, sterile filtrated thiamine hydrochloride stock solution (1 g/L) was added. Final thiamine concentrations were 0.002 mg/L, 0.02 mg/L, 0.1 mg/L, 0.2 mg/L, 2 mg/L, 10 mg/L and 20 mg/L.

### Cultivations

For all cultivations, precultures were made. The precultures were inoculated with a stock culture of the given *E. coli *SG13009 pKK233-2 clone and cultivated in the same cultivation vessel under the same cultivation conditions as the given main culture. Main cultures were inoculated with an inoculation volume of 1% (v/v) of the main culture volume.

To determine the BFD portion of total protein, the carboligation activity in crude extract and the optical density cultivations were conducted in 250 mL Erlenmeyer flasks filled with 25 mL buffered LB medium with 10 g/L glycerol. The cultures were grown at 37°C, a shaking diameter of 50 mm and a shaking frequency of 150 rpm.

The oxygen transfer rate (OTR) in shake flasks was measured online with a self made RAMOS (Respiratory Activity MOnitoring System) device, as described by Anderlei et al. [24]. The RAMOS cultivations were performed in modified 250 mL Erlenmeyer flasks (RAMOS flasks) as described by Anderlei and Büchs [25]. The applied RAMOS device allows to run up to 8 modified Erlenmeyer flasks in parallel. The cultures were grown in 10 or 25 mL medium (buffered LB medium with 10 g/L glycerol, TB medium or modified PanG mineral medium) at 37°C, a shaking diameter of 50 mm and a shaking frequency of 150, 320 or 400 rpm. No antifoaming agents were added to the medium during the cultivations with high shaking frequencies.

BFD expression was induced by adding IPTG stock solution (100 mM) resulting in a final IPTG concentration of 1 mM.

### Cell disruption

Cell disruption was applied as described by Losen et al. [26]. After centrifuging 3 mL culture medium for 15 min at 600 g, the cell pellets were frozen at -20°C and subsequently resuspended in extraction buffer (50 mM K_3_PO_4_, 5 mM MgSO_4_, 0.5 mM thiamine diphosphate (ThDP), pH 7). These samples were centrifuged for 10 min at 10000 g and the pellet was resuspended in extraction buffer with 1 mg/mL lysozyme. After incubation for 1.5 h at 30°C the samples were ultrasonicated for 5 min and centrifuged for 15 min at 10000 g. The supernatants were used to determine the BFD portion of total protein, the protein determination and the volumetric carboligation activity in crude extract.

### BFD portion of total protein

The portion of BFD in the cell extract was calculated by the quotient of the specific activity in the cell extract and the specific activity of purified BFD, as described by Losen et al. [26]. Determination of protein concentration was performed according to Bradford [27] using BSA for calibrations. Table 2 shows the specific decarboxylation activities that were applied.

**Table 2:** Specific decarboxylase activity of selected BFD-variants

BFD-variant	Specific decarboxylation activity [U/mg BFD]
wt	390

His281Ala	35

Leu476Gln	246

Leu476Pro	63

Leu476Pro-Ser181Thr	150

Ser181Thr	236

### Volumetric carboligation activity in crude extract

To determine the carboligation activity of the wild type BFD and the BFD-variants, cell extract was diluted 1/20 in reaction buffer (1.5 M ethanol, 50 mM K_3_PO_4_, 2.5 mM MgSO_4_, 0.1 mM ThDP). An equal volume of substrate solution (1 M acetaldehyde, 80 mM benzaldehyde) was added. After incubation for 30 min at 30°C, the reaction was stopped by heating for 2 min at 95°C. The amount of formed 2-hydroxy-1-phenyl-propanone (2-HPP) was measured using a HPLC system. Separation was performed on a RP8-column (Macherey & Nagel, Düren, Germany) using 0.5%/20% acetic acid/acetonitrile (v/v) as eluent. The flow rate was 1.1 mL/min.

### Cell density

To determine the cell density, the optical density was measured with a spectrophotometer (Uvikon 922 A, Kontron Instruments, Milano, Italy) at a wavelength of 600 nm. 10 mm cuvettes were applied. To keep the measurements in the linear range between 0.03 and 0.3, the samples were diluted with NaCl solution (9 g/L). Cuvettes containing only NaCl solution (9 g/L) were used as blanks. Furthermore, the optical density of sterile medium was subtracted from the measured optical density.

### Purification of selected BFD-variants

Purification of the wild type BFD and BFD-variants was performed as described for pyruvate decarboxylase [6] using potassium phosphate buffer (50 mM, pH 7.0) for Ni-NTA chromatography and potassium phosphate buffer (50 mM, pH 6.0) containing ThDP (0.5 mM) and MgSO_4 _(2.5 mM) as elution buffer for the subsequent gel chromatography. Lyophilised BFD-variants were stored at -20°C.

### Enzymatic synthesis in buffer

The initial carboligase activities were measured as described by Lingen et al. [4]. 10-50 μg purified wild type BFD or BFD-variant were incubated in 0.5 mL of 50 mM KPi, pH 7.0, containing 0.5 mM ThDP, 2.5 mM MgSO_4 _in the presence of 20, 40 or 60 mM benzaldehyde and 500 mM acetaldehyde for 30 min at 30°C. The enzymes were heat inactivated and the resulting 2-HPP formed was measured using an analytical HPLC system (Gynkotek, Germering, Germany) with an ultraviolet monitor (263 nm). Separation was performed on a 318-Hypersil column (C&S, Langerwehe, Germany) using 0.5%/20% acetic acid/acetonitrile (v/v) as eluent. The flow rate was 1.1 mL/min. The retention time of 2-HPP was 12.8 min.

### Stability of cofactor binding

The stability of cofactor binding was tested as described by Lingen et al. [4]. 0.1 mg/mL of the BFD-variants were incubated in potassium phosphate buffer without the cofactors ThDP and MgSO_4 _for 24 h. Then, 50 μL samples were removed and decarboxylase activity was determined. For this purpose, a coupled enzymatic test as described by Iding et al. was conducted [11]. An assay mixture was applied consisting of 100 μL benzoylformate solution (50 mM, pH 6.0), 100 μL NADH (3.5 mM), 50 μL horse liver alcohol dehydrogenase (HLADH) (10 U) and 700 μL potassium phosphate buffer (50 mM, pH 6.0). After mixing and incubating at 30°C, 50 μL BFD solution was added to initiate the reaction. The descending curve was examined at 340 nm and the linear slope was calculated from 0 to 90 s. The activity of the different BFD-variants was compared to the activity of the given BFD-variant under the same conditions but with 700 μL potassium phosphate buffer (50 mM, pH 6.0) additionally containing 0.5 mM thiamine diphosphate and 2.5 mM MgSO_4_. One unit is defined as the amount of enzyme that catalyses the decarboxylation of 1 μmol benzoylformate per minute at pH 6.0 and 30°C.

## Results and Discussion

**Figure 1:**
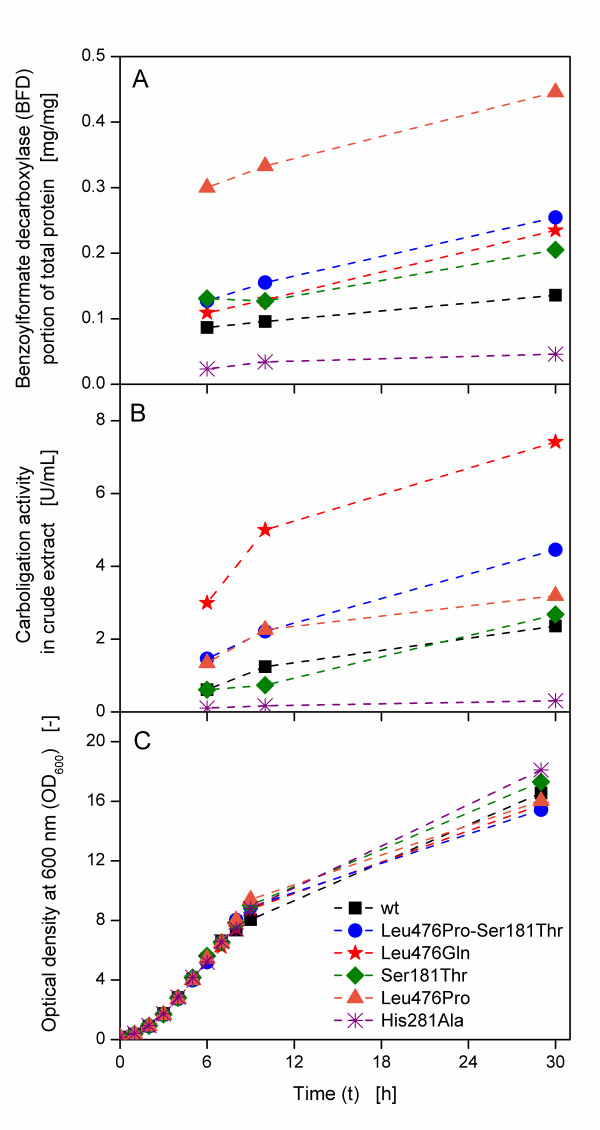
**(A) Benzoylformate decarboxylase (BFD) portion of total protein, (B) volumetric carboligation activity in crude extract and (C) optical density at 600 nm (OD**_**600**_**) of different BFD-variants**. Buffered LB medium (89 mM phosphate buffer) with 10 g/L glycerol; IPTG induction at 3 h; 250 mL shake flasks; filling volume: 25 mL; shaking frequency: 150 rpm; shaking diameter: 50 mm; temperature: 37°C.

Six clones of *E. coli *SG13009 pKK233-2, each carrying the gene for a different BFD-variant, were cultivated in 250 mL shake flasks containing 25 mL buffered LB medium with 10 g/L glycerol. The cultures were induced with IPTG after 3 h. Figure 1A depicts the BFD portion of total protein of the different clones during the cultivation for all applied clones. The BFD portion increases in all cases, however, the absolute level is completely different. This is astonishing, as host strain, plasmid, promoter construct and essentially the expressed gene are equivalent in all clones with only minor differences of one or two amino acids. His281Ala has the lowest portion with a value lower than 0.05 mg/mg after 30 h, followed by the wild type with about 0.1 mg/mg. The portion BFD-variants in all other clones is higher with a maximum portion of over 0.4 mg/mg for Leu476Pro. The volumetric carboligation activity is the value that is usually measured in a simple screening procedure. It is depicted in Figure 1B for the different BFD-variants. His281Ala has the lowest activity after 30 h with about 0.3 U/mL, followed by the wild type with ca. 2 U/mL. All other clones have higher activities, with Leu476Gln having the highest carboligation activity of about 8 U/mL. The optical densities (OD_600_) of the different clones are in the same range (Figure 1C). His281Ala has the highest OD_600 _of about 18 after 30 h and Leu476Pro-Ser181Thr the lowest OD_600 _of about 15.5.

**Figure 2:**
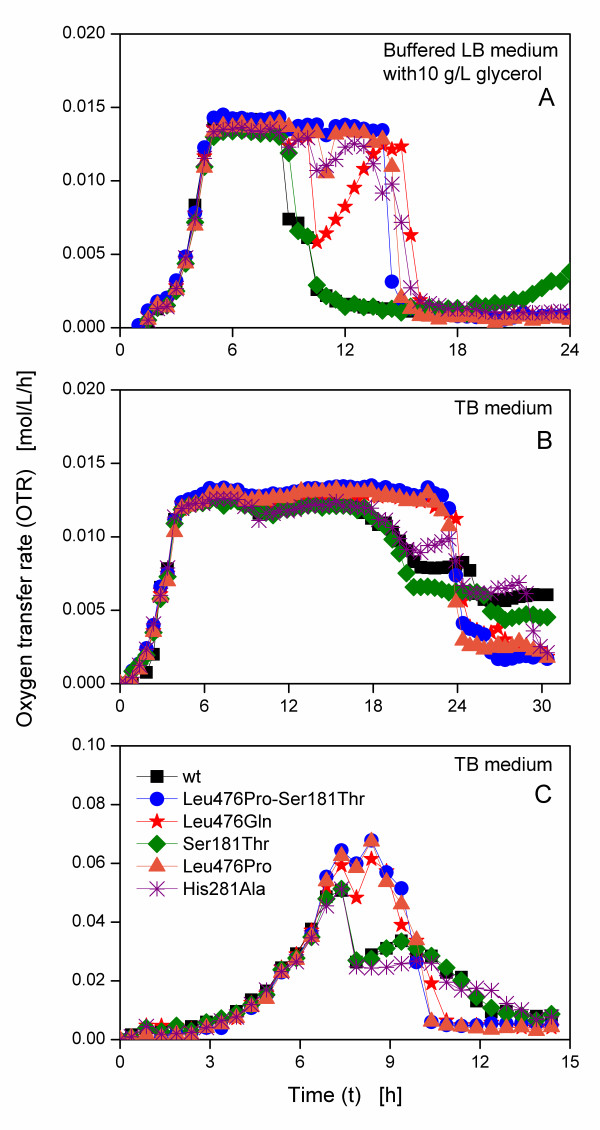
**Oxygen transfer rates of *E. coli *SG13009 pKK233-2 clones producing different BFD-variants**. Cultivation at 37°C, a shaking diameter of 50 mm and different cultivation conditions in modified 250 mL shake flasks. (A) Buffered LB medium (89 mM phosphate buffer) with 10 g/L glycerol; IPTG induction at 3 h; filling volume: 25 mL; shaking frequency: 150 rpm. (B) TB medium; IPTG induction at 3.5 h; filling volume: 25 mL; shaking frequency: 150 rpm. (C) TB medium; IPTG induction at 3.5 h; filling volume: 10 mL; shaking frequency: 400 rpm.

Figure 2A shows the corresponding OTR curves of the different *E. coli *SG13009 pKK233-2 clones cultivated in 250 mL RAMOS flasks containing 25 mL buffered LB medium with 10 g/L glycerol. Up to about 9 h, the curves of all clones are similar. After an exponential increase of OTR for 5 h to ca. 0.014 mol/L/h, a plateau caused by an oxygen limitation [25] follows. A low shaking frequency in combination with a relatively high filling volume leads to an insufficient oxygen availability. Afterwards, three different types of respiration behaviours are visible. For the clones Leu476Pro-Ser181Thr and Leu476Pro, the plateau lasts up to ca. 15 h, subsequently followed by a rapid decline, whereas the OTRs of the clone expressing the wild type BFD and Ser181Thr decline to 0.007 mol/L/h after only 10 h and reach 0.001 mol/L/h after 12 h. The OTRs of Leu476Gln and His281Ala decline after 10 h, too, but subsequently recover before they drop to about 0.001 mol/L/h after 16 h and 15 h, respectively.

Cultivation of the *E. coli *SG13009 pKK233-2 clones under the same conditions in TB medium results obviously in two different types of OTR curves (Figure 2B). Again, Leu476Pro-Ser181Thr and Leu476Pro reach a plateau of 0.013 mol/L/h after an exponential increase. This plateau is again due to an oxygen limitation. Its level is slightly lower than for LB medium, as TB is a richer medium resulting in lower oxygen solubility and diffusivity [28]. After 24 h, the OTRs drop. The OTR of the second type (wild type and Ser181Thr) also increase exponentially to 0.013 mol/L/h. However, they decline already after 18 h to about 0.06 mol/L/h. Further respiration activity at this lower level ensues before a second decline follows after ca. 25 h. In contrast to the first cultivation shown in Figure 2A, here, Leu476Gln and His281Ala behave like the first type and the second type, respectively.

To surely avoid an oxygen limitation, another experiment with a lower filling volume (10 mL) and higher shaking frequency (400 rpm) was conducted (Figure 2C). No plateau of the OTR was observed. Again, different types of OTR curves depending on the clones occurred. Up to about 7 h, all OTRs increase exponentially to 0.065 mol/L/h, yet the following respiration behaviours differ. The OTRs of the first type (Leu476Pro-Ser181Thr, Leu476Gln and Leu476Pro) slightly drop, before another increase until ca. 8.5 h follows. All three curves drop after 11 h. The curves of the second type (the clone expressing the wild type BFD, Ser181Thr and His281Ala), however, drop to ca. 0.025 mol/L/h after 8 h. The OTR of His281Ala remains at this level, while the curves of the clone expressing the wild type BFD and Ser181Thr recover to over 0.03 mol/L/h after 9.5 h. Subsequently, all three curves decline steadily.

From Figure 2A-C, three different groups of clones can be distinguished on basis of the OTR curves. The first group consists of Leu476Pro-Ser181Thr and Leu476Pro, which show typical OTR curves under oxygen limited and not oxygen limited conditions. In contrast, the OTR of the second type shows an earlier drop of OTR which is followed by further respiration activity on a lower level in TB medium. The third type (Leu476Gln and His281Ala) shows a variable behaviour, depending on the cultivation conditions. All these clones, having only very small differences in their genetic construction, behave surprisingly different in their metabolic activity, proving the differences that were already found in their expression properties shown in Figure 1.

**Figure 3:**
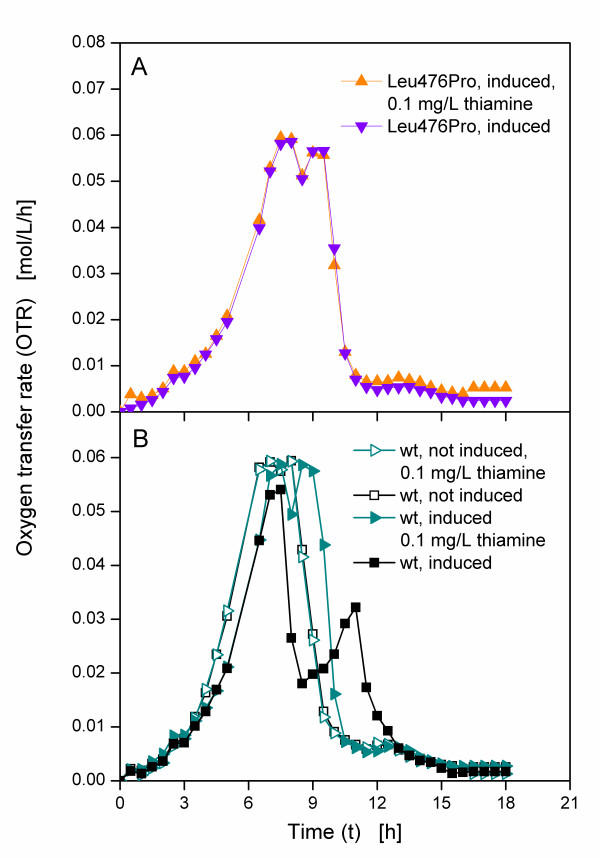
**Oxygen transfer rates of *E. coli *SG13009 pKK233-2 clones producing different BFD-variants in TB medium**. Modified 250 mL shake flasks; filling volume: 10 mL; shaking frequency: 320 rpm; shaking diameter: 50 mm; temperature: 37°C; induced cultures were induced after 3.5 h.

As the applied *E. coli *strain SG13009 is thiamine auxotroph and thiamine is needed for growth as well as a cofactor for BFD, it was supposed that thiamine caused this astonishing behaviour. However, it must be stressed that LB and TB media are usually applied without any supplementation of additional thiamine [21-23]. Thus, further cultivations were conducted in normal TB medium with additional thiamine supplementation. To test one clone of each type, Leu476Pro and the clone expressing the wild type BFD were chosen. While the first exhibited no early drop of respiration activity under all investigated culture conditions, the latter showed an early drop of respiration activity. Both clones were cultivated in TB medium with and without 0.1 mg/L additional thiamine. The Leu476Pro clone was induced after 3.5 h, resulting in OTR curves that are nearly identical, irrespective of thiamine addition (Figure 3A). The same cultivations were conducted with the clone expressing the wild type BFD, yet one culture in each medium was induced after 3.5 h and one was not induced. Figure 3B shows the OTRs of these cultures. Compared to the non-induced cultures, the induced cultures show a slower increase of OTR upon induction after 3.5 h due to the metabolic burden, the cultures are exposed to. That means, cellular resources are used for the production of BFD, thus reducing the growth of the cultures [29,30]. The curves of the non-induced cultures are nearly identical in both media, whereas the curves of the induced cultures differ. The OTR of the induced clone that expresses the wild type BFD in TB medium rises to about 0.05 mol/L/h after 8 h, drops to below 0.02 mol/L/h after 9 h and increases to 0.03 mol/L/h after 11 h, before it declines to nearly 0 mol/L/h after about 15 h. Up to 8 h the curve of the induced culture in TB medium with additional thiamine is similar, yet the following decline is smaller. The OTR only decreases to about 0.05 mol/L/h, before it rises again to approximately 0.06 mol/L/h after 9 h.

The addition of thiamine seems to have no effect on the growth of Leu476Pro. This clone shows no limitation in TB medium without additional thiamine and the OTR curves of Leu476Pro in TB medium with and without additional thiamine hardly differ. For the induced clone expressing the wild type BFD, the addition of thiamine to the medium leads to an OTR curve without early decrease. Apart from a slower increase due to the metabolic burden, the curve of the induced culture with additional thiamine is similar to the curves of the non-induced cultures. These results support the assumption that the early drop of OTR during the cultivation of the clone expressing the wild type BFD in TB medium without additional thiamine was caused by the level of thiamine concentration.

**Figure 4:**
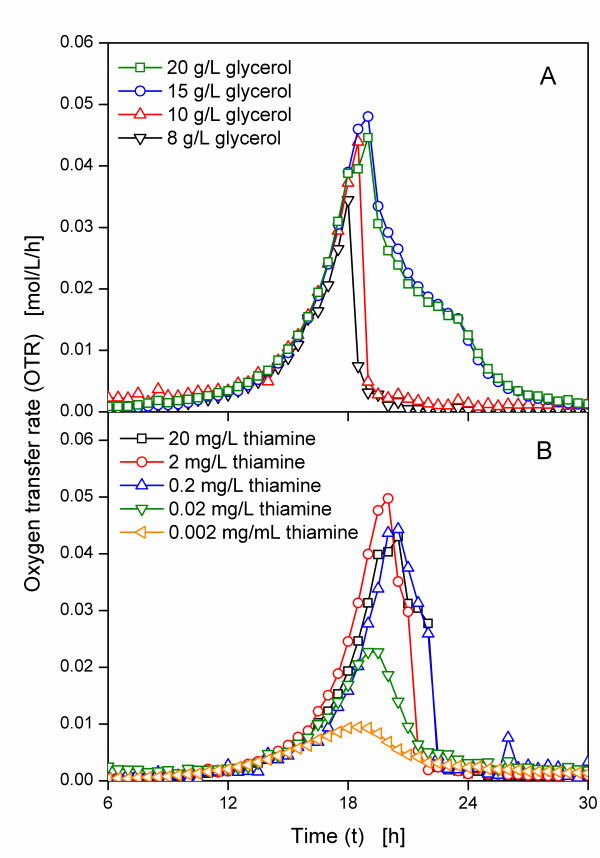
**Oxygen transfer rates of *E. coli *SG13009 pKK233-2 expressing wild type BFD in modified PanG mineral medium**. Modified 250 mL shake flasks; filling volume: 10 mL; shaking frequency: 320 rpm; shaking diameter: 50 mm; temperature: 37°C; all OTRs are shown from 6 h to enlarge the relevant part of the graphs. (A) Different initial glycerol concentration (10 mg/L thiamine). (B) Different initial thiamine concentration (10 g/L glycerol).

In complex medium thiamine is part of the complex compounds. Thiamine is heat sensitive, and, thus, might partially be degraded when the medium is autoclaved. Therefore, variances in the duration of autoclaving and the subsequent cooling may cause different thiamine concentrations in the medium. Additionally, the amount of thiamine in the complex compounds can vary, too, leading to an undefined thiamine concentration in the medium. A mineral medium was, thus, applied for further cultivations. To determine the required amount of glycerol and thiamine, the clone expressing the wild type BFD was cultivated in modified PanG mineral medium with different glycerol and thiamine concentrations. To avoid the problem of heat degradation of thiamine, sterile filtrated thiamine solution was added after the medium was autoclaved. Figure 4A shows the OTR curves of the clone expressing the wild type BFD in PanG medium with 10 mg/L thiamine and different glycerol concentration. The cultures in PanG medium with 8 g/L and 10 g/L glycerol are nearly identical and show OTR curves typical for batch cultures, with the culture in medium with 10 g/L glycerol having a higher maximum OTR of 0.045 mol/L/h. Whereas the first part of the OTR curves of the cultures with 20 g/L and 15 g/L glycerol is also identical up to about 20 h, the subsequent slow decline suggests another limitation [25], which was, however, not further investigated in this work. As this limitation does not occur in PanG medium with 10 g/L glycerol, this concentration was applied for further cultivations.

The thiamine concentration was varied between 0.002 mg/L and 20 mg/L (Figure 4B). The highest maximum OTR of 0.05 mol/L/h is obtained by the culture with 2 mg/L thiamine. Up to this concentration, increasing the thiamine concentration results in an increased maximum OTR. However, the addition of 20 mg/L thiamine leads to a lower maximum OTR of about 0.04 mol/L/h. While a concentration of 0.02 mg/L thiamine is sufficient to allow the growth of the applied clone, a concentration of 0.05 mg/L thiamine was selected for further cultivations to ensure a sufficient thiamine concentration during further cultivations with induction.

**Figure 5:**
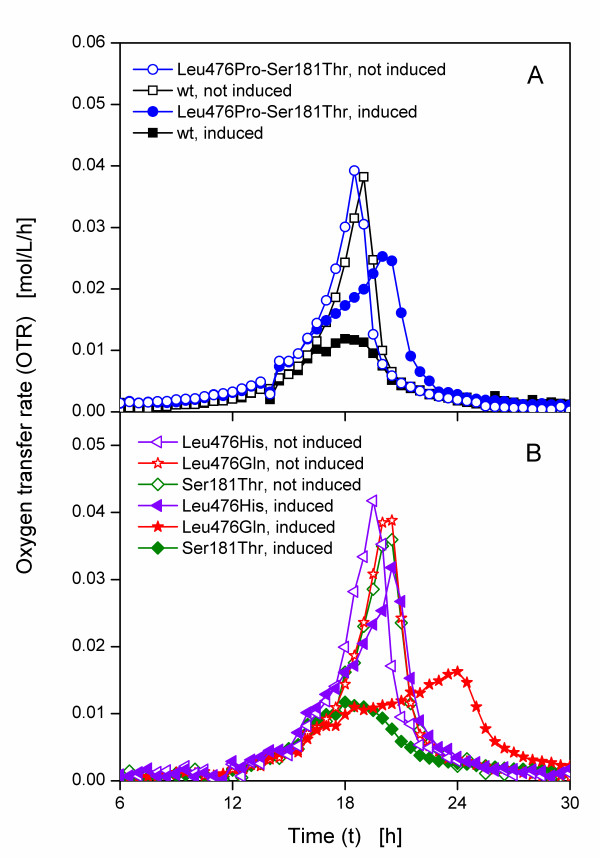
**Oxygen transfer rates of clones of *E. coli *SG13009 pKK233-2 expressing different BFD-variants in modified PanG mineral medium with 0.05 mg/L thiamine**. (A) wild type and Leu476Pro-Ser181Thr. (B) Leu476His, Leu476Gln and Ser181Thr. Modified 250 mL shake flasks; filling volume: 10 mL; shaking frequency: 320 rpm; shaking diameter: 50 mm; temperature: 37°C, induced cultures were induced after 16 h; all OTR are shown from 6 h to enlarge the relevant part of the graphs.

Five *E. coli *SG13009 pKK233-2 clones expressing different BFD-variants (Leu476Pro-Ser181Thr, wild type, Leu476His, Leu476Gln and Ser181Thr) were selected for cultivation in modified PanG mineral medium with 10 g/L glycerol and 0.05 mg/L thiamine. As mentioned before, Figure 2 indicates three different types of clones. Thus, Leu476Pro-Ser181Thr was selected as an example for the first type which exhibited a sharp drop of OTR after a relative long time. The clone expressing the wild type BFD and Ser181Thr belong to the second type with an earlier drop of OTR and a subsequent growth at a lower OTR. For the third type, which shows variable OTR depending on the cultivation conditions, Leu476Gln was chosen. One culture of each clone was induced after 16 h with IPTG and one was not induced. Figure 5A depicts the OTR curves of the clone expressing the wild type BFD and Leu476Pro-Ser181Thr. Without induction, the OTR curves of the clone expressing the wild type BFD and Leu476Pro-Ser181Thr have the same shape (Figure 5A). After reaching a maximum OTR of about 0.04 mol/L/h after 18 h the OTR drops to 0.005 mol/L/h after 21 h. In contrast, the OTR curves of the induced cultures differ. Upon induction after 16 h, the slope of the curves of Leu476Pro-Ser181Thr decreases in comparison to the not induced culture, probably explained by the metabolic burden. It reaches its maximum OTR of about 0.025 mol/L/h after 21 h. The curve of the clone expressing the wild type BFD, however, reaches a maximum OTR of only 0.01 mol/L/h after 18 h, followed by a decline to about 0.002 mol/L/h after 24 h.

The OTR curves of the clones expressing Leu476His, Leu476Gln and Ser181Thr without induction are also similar (Figure 5B). They show a maximum OTR of about 0.04 mol/L/h after ca. 18 h. For Leu476His, the OTR of the induced culture does not change in comparison to the not induced culture, but has a reduced maximum OTR of 0.03 mol/L/h and a decreased slope upon induction, which is again caused by the metabolic burden. For the clones Ser181Thr and Leu476Gln, however, the curves of the induced cultures vary strongly compared to the curves of the not induced cultures. With a maximum OTR of 0.01 mol/L/h, the OTR of the induced Ser181Thr culture is similar to the OTR of the clone expressing the wild type BFD shown in Figure 5A. After induction at 16 h, the OTR of Leu476Gln increases slowly to 0.015 mol/L/h after 24 h.

Regarding the induced cultures in Figure 5A and 5B, three different types of OTR curves can be distinguished. The first type (Leu476Pro-Ser181Thr and Leu476His) has a similar respiration activity as the non-induced cultures with a slightly reduced maximum OTR and a reduced initial increase of OTR upon induction due to the metabolic burden. In contrast to the OTR in complex medium (Figure 2C and 3B), the OTR of the second type (the clone expressing the wild type BFD and Ser181Thr) in mineral medium does not drop after a certain time, but shows a strongly decreased maximum OTR. Upon induction, the slope of the OTR curve of the third type (Leu476Gln) is drastically reduced, resulting in a maximum OTR of only 0.02 mol/L/h after 24 h.

**Figure 6:**
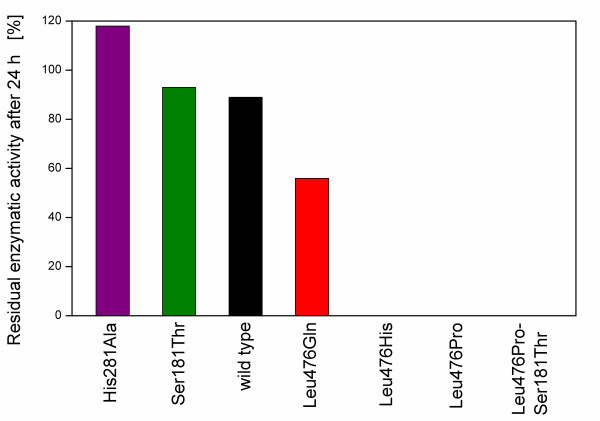
**Residual enzyme activity of different BFD-variants after incubation for 24 h in 50 mM KP**_**i**_**-buffer (pH 6.5) without thiamine**. The residual enzyme activity of the given variant refers to its activity in 50 mM KP_i_-buffer (pH 6.5) with thiamine (according to Lingen et al. [4]).

Figure 6 shows the residual enzyme activity of the BFD-variants after 24 h incubation in 50 mM KP_i_-buffer without thiamine. The BFD-variants His281Ala, Ser181Thr and the wild type BFD have about 100% residual enzyme activity after 24 h, whereas no activity is detected for Leu476His, Leu476Pro and Leu476Pro-Ser181Thr. In contrast, Leu476Gln neither maintains 100% of its enzyme activity after 24 h, nor looses it completely. After 24 h incubation, it retains a residual enzyme activity of about 60%. The reason for these differences in residual enzymatic activity might be the different cofactor binding strength of the variants. A high residual enzyme activity implies that the cofactor thiamine was not washed out because of the high cofactor binding strength of the given BFD-variants, whereas a low residual enzyme activity is caused by a low cofactor binding strength. After 24 h incubating in cofactor free buffer the cofactor, thiamine dissociated from Leu476His, Leu476Pro and Leu476Pro-Ser181Thr, therefore disabling their enzymatic activity.

According to Lingen et al. [4], Leu476 is located near, but not in the active centre of the enzyme and is involved in contacts between two of the four monomers the enzyme consists of [31]. The authors suppose it to have an influence on the cofactor binding strength, thus, by exchanging the amino acid at this position the cofactor binding strength of the enzyme can be altered. While the exchange of leucine by histidine or proline leads to a lower cofactor binding strength, exchanging it for glutamine also reduces the cofactor binding strength, but to a lesser degree. Contrarily, the replacement at the positions 181 and 281 does not reduce the cofactor binding strength.

These different cofactor binding strengths might explain the different OTR curves of the clones. Thiamine is a cofactor of BFD as well as of enzymes of the central carbon metabolism of *E. coli*, such as the pyruvate dehydrogenase complex (PDHc) [32]. The PDHc catalyses the formation of CO_2_, Acetyl-CoA (which is further utilised in the citric acid cycle) and NADH_2_^+ ^(which is used in the electron transport chain), from pyruvate, coenzyme A and NAD^+ ^[33]. Here, thiamine pyrophosphate (ThDP) is a cofactor of the subunit pyruvate dehydrogenase that deacetylates pyruvate under CO_2_-formation. ThDP is supposed to actively participate in the reaction of pyruvate dehydrogenase [34-37]. Thus, if thiamine is bound by other enzymes such as BFD and, in consequence, is not available for the PDHc, the reaction of the PDHc might be hampered, resulting in different OTR curves.

Besides its role as cofactor for several enzymes, thiamine in its phosphorylated form ThDP is also involved in the regulation of genes of the thiamine biosynthesis. In *E. coli*, the operons *thiCEFSGH*, *thiMD *and *tbpAthiPQ*, which code for enzymes of the thiamine biosynthesis and thiamine transporters, are regulated by ThDP [38]. ThDP binds at a conserved, untranslated RNA structure that is called *thi *box without involvement of protein cofactors. When thiamine is bound at this *thi *box riboswitch, the structure of the mRNA changes, masking the Shine-Dalgarno box and, thus, hindering the initiation of translation [39]. In consequence, the gene expression of the *thi *box riboswitch regulated genes is reduced [40] if thiamine is present in the medium, whereas in case of a lack of thiamine, the Shine-Dalgarno box is not masked and the gene expression is, thus, not hampered.

## Conclusions

**Figure 7:**
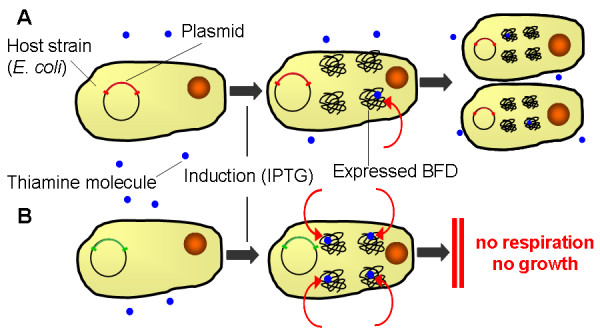
**Mechanistic reason for the behaviour of *E. coli *SG13009 pKK233-2 clones expressing different BFD-variants**. (A) BFD-variants with high cofactor binding strength: wild type, His281Ala, Ser181Thr, and partially Leu476Gln. As the thiamine is bound by these BFD-variants with high binding strength, it is not available in the medium and, thus, the growth of these strains is impaired. (B) BFD-variants with low cofactor binding strength: Leu476His, Leu476Pro, Leu476Pro-Ser181Thr.

This study showed that even a slight alteration of the amino acid composition of BFD has a surprisingly large effect on the expression and the metabolic activity of the applied host strain. The *E. coli *SG13009 pKK233-2 clones, each harbouring the gene for a different BFD-variant, not only express different amounts of the respective BFD-variant, but also show strongly varying OTR curves during cultivation. It was shown that the OTR curves of the clones are dependent on the thiamine availability in the medium which, in turn, is supposed to be dependent on the cofactor affinity of the expressed BFD-variants. Because thiamine is not only a cofactor for BFD, but also for other enzymes that are involved in the metabolism of *E. coli*, the different cofactor binding strengths and, thus, the availability of thiamine in the medium might be an explanation for the differences in the OTR curves. Leu476His, Leu476Pro and Leu476Pro-Ser181Thr are BFD-variants with low cofactor binding strength. As only a fraction of thiamine is bound by the BFD-variants, the medium contains enough thiamine to enable normal growth of these clones (Figure 7A). In contrast, the higher cofactor affinity of the wild type, His281Ala, Ser181Thr and partially Leu476Gln leads to impaired respiration and growth of the clones producing these BFD-variants, as the thiamine is presumably extracted from the medium and bound by the BFD-variants and is, hence, not available for growth (Figure 7B). The intermediate cofactor binding strength of Leu476Gln is a likely explanation for the OTR curves of this clone. Although it binds thiamine, the thiamine still can partially be dissociated, leading to variable respiration behaviour, depending on the cultivation conditions. Whereas this clone behaves like the first type in TB medium (Figure 2B and 2C), its OTR in modified PanG mineral medium is more similar to the second type. Its medium cofactor binding strength allows growth at a slower rate and a reduced maximum OTR compared to the first type, but for a longer time than the second type (Figure 5B).

As shown in this study, addition of thiamine to the medium can prevent the observed differing growth kinetics and is, thus, strongly recommended. While thiamine was identified as the cause for the differing growth kinetics in these experiments, other cultivations might be influenced by different effects. Unexpected phenomena such as the observed are hardly detectable in conventional screening processes. This might lead to wrong selection of enzyme variants, wrong assumptions about the optimal point of harvest and, ultimately, to wasted resources. To realise such hardly foreseeable effects influencing cultivation and expression of products, the application of on-line monitoring systems is, therefore, advised in screening processes.

While this study was conducted with the thiamine auxotroph strain *E. coli *SG13009, further studies should focus on prototrophic strains, such as *E. coli *BL21, to investigate, if the different binding strengths of the BFD-variants effect the OTR of these strains, too.

## Competing interests

The authors declare that they have no competing interests.

## Authors' contributions

TGP prepared the manuscript. JN performed the cultivation experiments. BF provided the applied strains and determined the cofactor binding strength of the different BFD-variants. MP planned the His281Ala variant and conducted structure-function studies of all variants on the basis of their 3D-structure. WT sponsored the project and assisted in the interpretation of the results. JB initiated the project, assisted with data analysis and manuscript preparation. All authors read and approved the final manuscript.
